# Factors Associated With Mild Cognitive Impairment in Patients With Type 2 Diabetes: A Cohort Study

**DOI:** 10.7759/cureus.28305

**Published:** 2022-08-23

**Authors:** Fatima Zahra Rhmari Tlemçani, Saloua Elamari, Imane Motaib, Soukaina Laidi, Najib Alidrissi, Samir Ahid, Asmaa Chadli

**Affiliations:** 1 Endocrinology, Diabetes, and Metabolism, Faculty of Medicine, Mohammed VI University of Health Sciences/Cheikh Khalifa International University Hospital, Casablanca, MAR; 2 Endocrinology, Diabetology, Metabolic Disease, and Nutrition, Mohammed VI University of Health Sciences, Casablanca, MAR; 3 Endocrinology, Diabetes, and Metabolism, Faculty of Medicine, Mohammed VI University of Health Sciences, Casablanca, MAR; 4 Orthopedics and Traumatology, Mohammed VI University of Health Sciences/Cheikh Khalifa International University Hospital, Casablanca, MAR; 5 Pharmacy, Mohammed VI University of Health Sciences, Casablanca, MAR; 6 Endocrinology, Diabetology, Metabolic Disease, and Nutrition, Mohammed VI University of Health Sciences/Cheikh Khalifa International University Hospital, Casablanca, MAR

**Keywords:** mini-mental state examination, complications of diabetes, cardiovascular risk factors, cognitive impairment, diabetes

## Abstract

Background

Cognitive dysfunction is increasingly recognized as an important comorbidity of diabetes mellitus (DM).

Objective

The purpose of this study was to determine the prevalence and predictors of cognitive decline in individuals with type 2 diabetes mellitus (T2DM).

Methods

This cohort study included patients with type 2 diabetes mellitus aged between 40 and 75 years and with a duration of the evolution of diabetes that is greater than five years admitted in endocrinology consultation of the Sheikh Khalifa ibn Zaid Hospital in Casablanca, Morocco. For each patient, we collected clinical characteristics and biological assessments. All subjects provided screening test results as defined by the Mini-Mental State Examination (MMSE).

Results

We included a total of 100 patients with diabetes between May and September 2021. The median age of the patients was 65 years (interquartile range (IQR): 59-70 years), 65% were males, and the median duration of diabetes was 15 years (IQR: 9-20 years). The most common cardiovascular risk factors (CVRFs) were hypertension (72.7%) and dyslipidemia (53%). The most common complications of diabetes were peripheral neuropathy (50%), diabetic retinopathy (DR) (39%), peripheral artery disease (33%), and coronary artery disease (27%). Cognitive impairment was present in 47.5% of our patients. For the multivariate analysis, we found that the decrease in the MMSE score is associated with the increase in age (p-value = 0.004) and the occurrence of diabetic retinopathy (p-value < 0.001), dyslipidemia (p-value = 0.006), and elevated creatinine (p-value < 0.001).

Conclusion

It is necessary to consider the cognitive decline of patients with diabetes as one of the most important complications of this disease because of its impact on the evolution and compliance of these patients.

## Introduction

There is increasing evidence that patients with type 2 diabetes mellitus (T2DM) are predisposed to cognitive decline leading to dementia [1]. Up until now, the prevalence of diabetes and cognitive impairment are very similar, suggesting an overlap in the risk factors associated with the two diseases [2,3]. In the United State of America (USA), data from a prominent veterans' registry revealed that the prevalence of dementia and cognitive impairment associated with diabetes was 13.1% for those aged 65-74 years [4]. As a result, cognitive decline is increasingly recognized by patients with diabetes and their physicians. This complication remains less explored and treated than other complications of diabetes [5].

Patients with mild cognitive impairment (MCI) have an increased risk of developing cardiovascular risk factors (CVRFs) [6]. The impact of CVRFs, such as diabetes mellitus, dyslipidemia, obesity, and hypertension, on cognition exists [7,8]. However, the physiopathology of CVRFs in patients with mild cognitive impairment is still unclear.

Furthermore, managing the diabetic condition requires cognitive functions, such as attention, memory, planning, and calculating. Impaired cognitive function may threaten the patient's ability to perform self-monitoring of glucose and thus the ability to self-manage their diseases [9].

In this study, we investigated the link between cardiovascular risk factors in patients with type 2 diabetes and cognitive disorders that could help detect subjects who have a higher risk of developing cognitive decline in our Moroccan society.

## Materials and methods

Study design

This observational cohort study included all patients with T2DM aged between 40 and 75 years and with a duration of the evolution of diabetes that is greater than five years admitted in endocrinology consultation of the Sheikh Khalifa ibn Zaid Hospital in Casablanca, Morocco, between February 2021 and February 2022. We excluded patients with a history of ischemic stroke and depression, on antiepileptic drugs, with glycosylated hemoglobin (HbA1c) < 4%, and who are older than 75 years.

Clinical assessment

For each patient, we collected data related to his/her demographic characteristics involving gender and age, comorbidities (hyperuricemia, hypertension, amputation, and cardiovascular diseases), microvascular complications (diabetic nephropathy, peripheric neuropathy, and diabetic neuropathy), macrovascular complications (coronary heart disease and peripherical arterial disease), and biological assessments (HbA1c, creatinine, low-density lipoprotein (LDL) cholesterol, uric acid, and urinary microalbuminuria). Finally, all subjects provided Mini-Mental State Examination (MMSE) results.

Predicting variables

The different complications were acknowledged using standard definitions. Peripheral sensory neuropathy was detected by testing pain evoked by light touch according to Michigan Neuropathy Screening Instrument scores [10]. Nephropathy was defined as a urinary albumin/creatinine ratio of more than 3 mg/mmol in an early-morning urine sample [11]. Diabetic retinopathy (DR) was observed in patients who reported the occurrence of proliferative diabetic retinopathy at baseline and underwent standardized eye examinations. Coronary heart disease was defined by the informed history of angina, coronary artery bypass grafting, or angioplasty myocardial infarction [12]. Peripheral arterial disease was considered present when arterial ultrasonic examination of the lower limbs or arteriography was performed previously and showed atheromatous plaques and/or arterial stenosis [11].

Cognitive function

Global cognition was defined using the Mini-Mental State Examination (MMSE) adapted to the Maghrebi population, a widely used test in clinical and research settings, taking into account education level [13]. A positive screening test was defined by an MMSE score of <27/30 [14].

Ethical considerations

The ethics board of Mohammed VI University of Health Sciences approved our study. Data confidentiality and patient anonymity were maintained at all stages of the study (data file number: CERB/UM6SS/11/21). We deleted patient-identifying information before analyzing the database. Informed consent was obtained for all patients before inclusion.

Data analysis

Data were analyzed using JAMOVI version 1.6.23.0 [15]. Descriptive analysis was performed for continuous variables using medians with interquartile ranges (IQRs) and categorical variables using percentages. The difference in the cognitive status was expressed using the nonparametric Mann-Whitney U test on the equality of medians for continuous variables and Fisher's exact test and chi^2^ test for categorical variables.

Univariate linear regression was presented to study the link between all available variables, as described above, and cognitive impairment. Multivariate linear regression was performed to study the association between cognitive impairment and all available variates. Since cognitive impairment might be highly correlated with many variables, particularly complications of diabetes, we used a stepwise multivariate model based on a bidirectional elimination, with a p-value of <0.2 and including all significant control variables as found in the univariate analysis.

## Results

A total of 100 patients with diabetes who consulted at the endocrinology consultation of Sheikh Khalifa ibn Zaid Hospital during the period defined above were included in our study. The median age of patients was 65 years (IQR: 59-70 years); the male/female ratio was 1.8, and the median duration of diabetes in the patients was 15 years (IQR: 9-20 years). The most common cardiovascular risk factors were hypertension (72.7%) and dyslipidemia (53%). The most common complications of diabetes in our sample of patients were peripheral neuropathy (50%), diabetic retinopathy (39%), peripheral artery disease (33%), and coronary artery disease (27%). Concerning the biochemical analysis of patients in the study, the median HbA1c was 8% (IQR: 7.1%-9.13%), the median LDL cholesterol was 0.9 mg/L (IQR: 0.71-1.2 mg/L), microalbuminuria was positive in 17% of the patients, the median creatinine was 7.25 mg/L (IQR: 6.3-9.93 mg/L), the median uretic acid was 53 mg/L (IQR: 36.5-63 mg/L), and the median body mass index (BMI) was 26.5 kg/m^2^ (IQR: 23-30 kg/m^2^). Cognitive impairment as defined by an MMSE score < 27/30 was present in 47.5% of our patients (Table 1).

**Table 1:** Characteristics of the study population IQR: interquartile range; BMI: body mass index; HbA1c: glycosylated hemoglobin; LDLc: low-density lipoprotein cholesterol

Variables	Values
Age, median (IQR), years	65 (59-70)
Males, n (%)	65 (65)
Duration of diabetes, median (IQR), years	15 (9-20)
Tobacco use, n (%)	18 (18)
Dyslipidemia n (%)	53 (53)
BMI, median (IQR), kg/m^2^	26.50 (23-30)
Hypertension, n (%)	72 (72)
Hyperuricemia, n (%)	9 (9)
Diabetic retinopathy, n (%)	39 (39)
Diabetic nephropathy, n (%)	17 (17)
Peripheric neuropathy, n (%)	50 (50)
Coronary artery disease, n (%)	27 (27)
Peripheral artery disease, n (%)	30 (33)
Podiatric grade, n (%)	
0	64 (64)
1	14 (14)
2	10 (10)
3	12 (12)
Amputation, n (%)	6 (6)
HbA1c, median (IQR), %	8 (7.10-9.13)
LDLc, median (IQR), (g/L)	0.90 (0.71-1.20)
Positive microalbuminuria, n (%)	17 (17)
Creatinine, median (IQR), mg/L	7.25 (6.30-8.93)
Uric acid, median (IQR), mg/L	53 (36.50-63)

Concerning the status of Mini-Mental State Examination scores in our sample of patients, there was a significant difference in age (p-value < 0.001), dyslipidemia (p-value = 0.022), microvascular complications concerning diabetic nephropathy (p-value = 0.001), diabetic retinopathy (p-value < 0.001), macrovascular complications including coronary heart disease (p-value = 0.029) and peripheral artery disease (p-value = 0.046). There was also a significant difference in terms of certain biochemical analyses including LDL cholesterol (p-value = 0.018), positive microalbuminuria (p-value = 0.001), and creatinine (p-value < 0.001) (Table 2).

**Table 2:** Comparison of the study population as per their cognitive status IQR: interquartile range; BMI: body mass index; AOMI: acute and old myocardial infarction combined; HbA1c: glycosylated hemoglobin; LDLc: low-density lipoprotein cholesterol

Variables	Cognitive impairment (n = 52) (52%)	No cognitive impairment (n = 47) (47%)	P-value
Age, median (IQR), years	69.50 (60-73)	63 (58.50-65.50)	<0.05
Males, n (%)	35 (35)	30 (30)	0.71
Duration of diabetes, median (IQR), years	15 (10-20)	14 (8-19)	0.25
Tobacco use, n (%)	10 (10)	8 (8)	0.81
Dyslipidemia n (%)	33 (33)	19 (19)	0.02
BMI median (IQR), kg/m^2^	26 (22-30)	27 (23-30)	0.98
Hypertension, n (%)	41 (41)	30 (30)	0.06
Hyperuricemia, n (%)	7 (7)	2 (2)	0.10
Diabetic retinopathy, n (%)	32 (32)	7 (7)	<0.05
Diabetic nephropathy, n (%)	15 (15)	2 (2)	<0.05
Peripheric neuropathy, n (%)	28 (28)	21 (21)	0.36
Coronary artery disease, n (%)	19 (19)	8 (8)	0.02
AOMI, n (%)	22 (22)	11 (11)	0.04
Podiatric grade, n (%)			0.20
0	32 (32)	32 (32)	
1	10 (10)	4 (4)	
2	3 (3)	7 (7)	
3	7 (7)	4 (4)	
Amputation, n (%)	3 (3)	3 (3)	1.00
HbA1c, median (IQR), %	8.54 (7.18-10)	8 (6.95-8)	0.064
LDLc, median (IQR), g/L	1.03 (0.83-1.27)	0.80 (0.66-1.16)	0.01
Positive microalbuminuria, n (%)	15 (15)	2 (2)	<0.05
Creatinine, median (IQR), mg/L	8.35 (6.95-12.30)	7 (6-8.10)	<0.05
Uric acid, median (IQR), mg/L	55.9 (47.50-65.50)	44.50 (34-63)	0.11

Regarding univariate analysis (Table 3), we observed that cognitive impairment was associated with age (ß: -0.171; 95% CI: -0.251 to -0.090) (p-value < 0.001), duration of diabetes (ß: -0.157; 95% CI: -0.251 to -0.090) (p-value = 0.009), cardiovascular risk factors including hypertension (ß: -2.76; 95% CI: -4.68 to -0.836) (p-value = 0.005) and dyslipidemia (ß: -2.3; 95% CI: -4.10 to -0.682) (p-value = 0.007), complications of diabetes including diabetic retinopathy (ß: -4.82; 95% CI: -6.35 to - 3.29) (p-value < 0.001), diabetic nephropathy (ß: -5.61; 95% CI: -7.67 to -3.55) (p-value < 0.001), peripheric neuropathy (ß: -1.98; 95% CI: -3.71 to -0.253) (p-value = 0.025), coronary heart disease (ß: -3.000; 95% CI: -4.90 to -1.11) (p-value = 0.002), peripheral artery disease (estimate: -2.23; 95% CI: -4.05 to -0.402) (p-value = 0.017), and podiatric grade 3 (ß: -4.004; 95% CI: -6.79 to -1.21) (p-value = 0.005). Cognitive impairment was associated to biochemical analysis including the rate of HbA1c (ß: -1.06; 95% CI: -1.70 to -0.432) (p-value = 0.001), positive microalbuminuria (ß: -5.61; 95% CI: -7.67 to -3.55) (p-value < 0.001), and the rate of creatinine (ß: -0.501; 95% CI: -0.686 to -0.317) (p-value < 0.001).

For the multivariate analysis (Table 3), we found that the decrease in the MMSE score is associated to the increase in age (ß: -0.0975; 95% CI: -0.163 to -0.00324) (p-value = 0.004), the occurrence of diabetic retinopathy (ß: -1.825; 95% CI: -3.109 to -0.541) (p-value < 0.001), and dyslipidemia (ß: -3.423; 95% CI: -4.793 to -2.054) (p-value < 0.001).

**Table 3:** Analysis of factors associated with cognitive impairment in patients with diabetes BMI: body mass index; CI: confidence interval; HbA1c: glycosylated hemoglobin; LDLc: low-density lipoprotein cholesterol

Variables	Univariate analysis coefficient β (95% CI)	Multivariate analysis coefficient β (95% CI)	Multivariate analysis stepwise coefficients β (95% CI)
Age (years)	-0.17 (-0.25, -0.090)	-0.14 (-0.35, 0.060)	-0.09 (-0.16, -0.03)
Male (%)	-0.40 (-2.27, 1.46)	0.014 (-0.27, 0.30)	
Duration of diabetes (years)	- 0.15 (-0.27, -0.04)	-0.05 (-3.39, 3.28)	
Tobacco use (%)	0.19 (-2.10, 2.50)		
Hypertension (%)	-2.76 (-4.68, -0.83)	0.17 (-3.08, 3.43)	
Hyperuricemia (%)	-2.56 (-5.61, 0.49)	-2.14 (-6.88, 2.59)	
Dyslipidemia (%)	-2.39 (-4.10, -0.68)	-1.89 (-5.09, 1.30)	-1.82 (-3.10, -0.54)
Diabetic retinopathy (%)	-4.82 (-6.35, -3.29)	-0.91 (-4.88, 3.04)	-3.42 (-4.79, -2.05)
Diabetic nephropathy (%)	-5.61 (-7.67, -3.55)	-1.69 (-9.32, 5.94)	
Peripheric neuropathy (%)	-1.98 (-3.71, -0.25)	-2.27 (-4.89, 0.34)	
Coronaropathy heart disease (%)	-3.00 (-4.90, -1.11)	-2.84 (-6.51, 0.83)	
Peripheric arteriopathy (%)	-2.23 (-4.05, -0.40)	0.26 (-3.01, 3.53)	
Amputation (%)	-0.78 (-5.28, 3.72)		
Podiatric grade			
1-0	-0.99 (-3.52, -1.52)	1.46 (-3.63, 6.56)	
2-0	-0.54 (-3.45, 2.37)	2.88 (-2.45, 8.23)	
3-0	-4.00 (-6.79, -1.21)	0.89 (-4.79, 6.58)	
HbA1c (%)	-1.06 (-1.70, -0.432)	-0.18 (-0.84, 0.48)	
LDLc (g/L)	-0.91 (-3.40, 1.56)		
Positive microalbuminuria (%)	-5.61 (-7.67, -3.55)		
Creatinine (mg/L)	-0.50 (-0.68, -0.31)	-0.00 (-0.08, 0.072)	-0.34 (-0.50, -0.18)
Uric acid (mg/L)	-0.05 (-0.13, 0.017)		

## Discussion

Prevalence

The prevalence of cognitive impairment was 47.5% in our patients; it could be explained by the elevated median age of our population. These findings are in agreement with other investigations [1]. In their study that involved 169 patients with type 2 diabetes whose cognitive status was assessed using Mini-Mental State Examination (MMSE), Świątoniowska-Lonc et al. [9] found that 56.8% of patients had cognitive impairment (MMSE < 27).

Predictors of cognitive decline in patients with diabetes

Age

In the present study, we found that patients with cognitive decline compared with those with normal cognition were older. These findings are in agreement with other investigations such as the study of Messier et al. [16], which reported deficits in measures of attention due to subcortical and white matter changes [17]. Among other small vessel cerebrovascular diseases, the risk factors glucose intolerance and hyperinsulinemia [18] and type 2 diabetes [19] have been shown to be associated with white matter disease. Metabolic disturbances accompanying insulin resistance (IR) disrupt not only the functioning of the liver, muscles, and adipose tissue but also the brain [20,21]. Additionally, recent studies have demonstrated that peripheral IR results in loss of brain function, which indicates a strong relationship between metabolic disturbances and cerebral degeneration [20].

Chronic Kidney Disease (CKD)

The independent effect of CKD severity on cognitive decline has not been fundamentally studied, even if both CKD and dementia share common risk factors. One of the principal findings of this study was that progressive CKD with increasing creatinine (taking into account that creatinine is one of the indicators of renal failure depending on age and nutritional status) is associated with poorer cognitive performance measured using Mini-Mental State Examination. The Japanese study by Lee et al. [22] investigated the link between CKD and cognitive decline in patients without dementia. They demonstrated that lower GFR is associated with poorer cognitive function. The mechanism of cognitive decline in patients with decreased renal function is still ambiguous [23]. According to Ikram et al. [24], it is due to endothelial dysfunction and inflammation, the markers of which are elevated independently of cardiovascular risk factors.

Diabetic Retinopathy

In our patient sample, we found that the onset of diabetic retinopathy (DR) was associated with increased cognitive decline. Ding et al. [25] studied a population of 1,046 patients with type 2 diabetes in Edinburgh. They found that, in this representative population of elderly people with type 2 diabetes, increasing severity of DR was associated with poorer overall cognitive ability [26,27]. Because of the considerable homology between the retinal and cerebral microvasculature, retinal vascular changes are likely to provide an indirect marker of concomitant changes in the cerebral microvasculature.

Dyslipidemia

The impact of dyslipidemia on cognitive decline in patients with diabetes is unclear. In our study, the level of cognitive impairment was influenced by the co-occurrence of diabetes and dyslipidemia and possibly by the association of other cardiovascular risk factors. Vintimilla et al. [7] studied the impact of cardiovascular risk factors on cognitive status in a Mexican cohort of 515 patients using several tests to measure global cognition of the MMSE. They found that when diabetes and dyslipidemia were present, this affected the ability to think, reason, and remember [28].

Currently, we know that the apolipoprotein E4 is detected in astrocytes and microglial cells and is also perceived in vascular endothelial cells, neurons, and neuritic activities. *ApoE4* gene plays a critical role in lipid metabolism and brain physiology and is also associated with an increased risk for cognitive decline. De Strooper and Karran [29] demonstrated that widespread deposition of apolipoprotein β (Aβ ) in Alzheimer's disease (AD) is both the cause and consequence of vascular pathology. The pathogenic impact of increased Aβ deposits with *ApoE4* in MCI could provide a viable genetic factor for detecting the risk of cognitive decline in patients with type 2 diabetes [30].

Impact of cognitive impairment on adherence to treatment

The cognitive impairment in patients with diabetes impairs verbal memory and psychomotor skills [31,32]. Tasks related to diabetes self-management involve multiple skills, such as memory, attention, planning, and calculation [33], and have an impact on blood glucose control [34]. In a Polish study that analyzed the impact of cognitive impairment on self-monitoring of blood glucose, they found that the self-monitoring in patients with cognitive impairment is less performant than in other patients [9].

Treatment of cognitive impairment in patients with diabetes

The search for innovative treatments to prevent cognitive impairment and improve cognition in people with mild cognitive impairment or Alzheimer's disease is ongoing. Several avenues have led to a dead end; other approaches seem promising [35]. For patients with diabetes, a recent large registry study of veterans with T2DM under 75 years of age found that metformin use was associated with a lower risk of subsequent dementia than sulfonylurea use while controlling for known confounders [36]. Perhaps drug safety studies should include neurological safety as well, in addition to cardiovascular safety.

Limitations and strength

This study has several strengths and limitations. The limitations of our study are the sample size and the occurrence of selection bias due to the realization of the study in a third-level hospital. We did not analyze the impact of medication use on diabetes, hypertension, and dyslipidemia. Finally, it would be better to consider the glomerular filtration rate as an indicator of CKD, because it is a more objective parameter than creatinine. The strengths of this study are that it includes well-typed MCI subjects, the use of neuropsychological tests that have been standardized for the Moroccan population, and its originality due to the fact that we still do not know everything about cognitive impairment in patients with diabetes.

## Conclusions

Recommendations for the management of people with diabetes who have been diagnosed with cognitive impairment are currently based primarily on expert opinion and limited to elderly populations. Although the rationale for these recommendations is clear, it is evident that additional multicentric studies are needed for the optimal management of these individuals. The age and risk factor profile increase considerably the incidence of dementia in patients with diabetes older than 60 years, and it may be appropriate to adapt the screening range based on the patient's cardiovascular risk factors and risk for dementia. Furthermore, regarding the prevention component, it is necessary to select appropriate screening tests to use in patients with type 2 diabetes.

## References

[1] Wong RH, Scholey A, Howe PR (2014). Assessing premorbid cognitive ability in adults with type 2 diabetes mellitus--a review with implications for future intervention studies. Curr Diab Rep.

[2] Biessels GJ, Despa F (2018). Cognitive decline and dementia in diabetes mellitus: mechanisms and clinical implications. Nat Rev Endocrinol.

[3] Arnold SE, Arvanitakis Z, Macauley-Rambach SL (2018). Brain insulin resistance in type 2 diabetes and Alzheimer disease: concepts and conundrums. Nat Rev Neurol.

[4] Feil DG, Rajan M, Soroka O, Tseng CL, Miller DR, Pogach LM (2011). Risk of hypoglycemia in older veterans with dementia and cognitive impairment: implications for practice and policy. J Am Geriatr Soc.

[5] Dolan C, Glynn R, Griffin S (2018). Brain complications of diabetes mellitus: a cross-sectional study of awareness among individuals with diabetes and the general population in Ireland. Diabet Med.

[6] van den Berg E, Kloppenborg RP, Kessels RP, Kappelle LJ, Biessels GJ (2009). Type 2 diabetes mellitus, hypertension, dyslipidemia and obesity: a systematic comparison of their impact on cognition. Biochim Biophys Acta.

[7] Vintimilla R, Balasubramanian K, Hall J, Johnson L, O'Bryant S (2020). Cardiovascular risk factors, cognitive dysfunction, and mild cognitive impairment. Dement Geriatr Cogn Dis Extra.

[8] Kloppenborg RP, van den Berg E, Kappelle LJ, Biessels GJ (2008). Diabetes and other vascular risk factors for dementia: which factor matters most? A systematic review. Eur J Pharmacol.

[9] Świątoniowska-Lonc N, Polański J, Tański W, Jankowska-Polańska B (2021). Impact of cognitive impairment on adherence to treatment and self-care in patients with type 2 diabetes mellitus. Diabetes Metab Syndr Obes.

[10] Ang L, Cowdin N, Mizokami-Stout K, Pop-Busui R (2018). Update on the management of diabetic neuropathy. Diabetes Spectr.

[11] American Diabetes Association (2021). 11. Microvascular complications and foot care: standards of medical care in diabetes-2021. Diabetes Care.

[12] Saraste A, Knuuti J (2020). ESC 2019 guidelines for the diagnosis and management of chronic coronary syndromes : recommendations for cardiovascular imaging. Herz.

[13] Pezzotti P, Scalmana S, Mastromattei A, Di Lallo D (2008). The accuracy of the MMSE in detecting cognitive impairment when administered by general practitioners: a prospective observational study. BMC Fam Pract.

[14] Arevalo-Rodriguez I, Smailagic N, Roqué-Figuls M (2021). Mini-Mental State Examination (MMSE) for the early detection of dementia in people with mild cognitive impairment (MCI). Cochrane Database Syst Rev.

[15] (2021). jamovi. http://www.jamovi.org/.

[16] Messier C, Tsiakas M, Gagnon M, Desrochers A (2010). Effect of age and glucoregulation on cognitive performance. J Clin Exp Neuropsychol.

[17] Bronge L (2002). Magnetic resonance imaging in dementia. A study of brain white matter changes. Acta Radiol Suppl.

[18] Feskens EJ, Kromhout D (1992). Glucose tolerance and the risk of cardiovascular disease: the Zutphen Study. J Clin Epidemiol.

[19] DeCarli C, Murphy DG, Tranh M (1995). The effect of white matter hyperintensity volume on brain structure, cognitive performance, and cerebral metabolism of glucose in 51 healthy adults. Neurology.

[20] Pugazhenthi S, Qin L, Reddy PH (2017). Common neurodegenerative pathways in obesity, diabetes, and Alzheimer's disease. Biochim Biophys Acta Mol Basis Dis.

[21] Heni M, Kullmann S, Preissl H, Fritsche A, Häring HU (2015). Impaired insulin action in the human brain: causes and metabolic consequences. Nat Rev Endocrinol.

[22] Lee S, Shimada H, Park H (2015). The association between kidney function and cognitive decline in community-dwelling, elderly Japanese people. J Am Med Dir Assoc.

[23] Li J, Wang YJ, Zhang M (2011). Vascular risk factors promote conversion from mild cognitive impairment to Alzheimer disease. Neurology.

[24] Ikram MA, Vernooij MW, Hofman A, Niessen WJ, van der Lugt A, Breteler MM (2008). Kidney function is related to cerebral small vessel disease. Stroke.

[25] Ding J, Strachan MW, Reynolds RM (2010). Diabetic retinopathy and cognitive decline in older people with type 2 diabetes: the Edinburgh Type 2 Diabetes Study. Diabetes.

[26] Patton N, Aslam T, Macgillivray T, Pattie A, Deary IJ, Dhillon B (2005). Retinal vascular image analysis as a potential screening tool for cerebrovascular disease: a rationale based on homology between cerebral and retinal microvasculatures. J Anat.

[27] Kwa VI, van der Sande JJ, Stam J, Tijmes N, Vrooland JL (2002). Retinal arterial changes correlate with cerebral small-vessel disease. Neurology.

[28] (2021). Verywell Health: Administration, scoring, and interpretation of the trail making test. https://www.verywellhealth.com/dementia-screening-tool-the-trail-making-test-98624.

[29] De Strooper B, Karran E (2016). The cellular phase of Alzheimer's disease. Cell.

[30] Yuan XY, Wang XG (2017). Mild cognitive impairment in type 2 diabetes mellitus and related risk factors: a review. Rev Neurosci.

[31] Moheet A, Mangia S, Seaquist ER (2015). Impact of diabetes on cognitive function and brain structure. Ann N Y Acad Sci.

[32] Kodl CT, Seaquist ER (2008). Cognitive dysfunction and diabetes mellitus. Endocr Rev.

[33] Biessels GJ, Deary IJ, Ryan CM (2008). Cognition and diabetes: a lifespan perspective. Lancet Neurol.

[34] Thabit H, Kennelly SM, Bhagarva A, Ogunlewe M, McCormack PM, McDermott JH, Sreenan S (2009). Utilization of Frontal Assessment Battery and Executive Interview 25 in assessing for dysexecutive syndrome and its association with diabetes self-care in elderly patients with type 2 diabetes mellitus. Diabetes Res Clin Pract.

[35] Andrade C, Radhakrishnan R (2009). The prevention and treatment of cognitive decline and dementia: an overview of recent research on experimental treatments. Indian J Psychiatry.

[36] Orkaby AR, Cho K, Cormack J, Gagnon DR, Driver JA (2017). Metformin vs sulfonylurea use and risk of dementia in US veterans aged ≥65 years with diabetes. Neurology.

